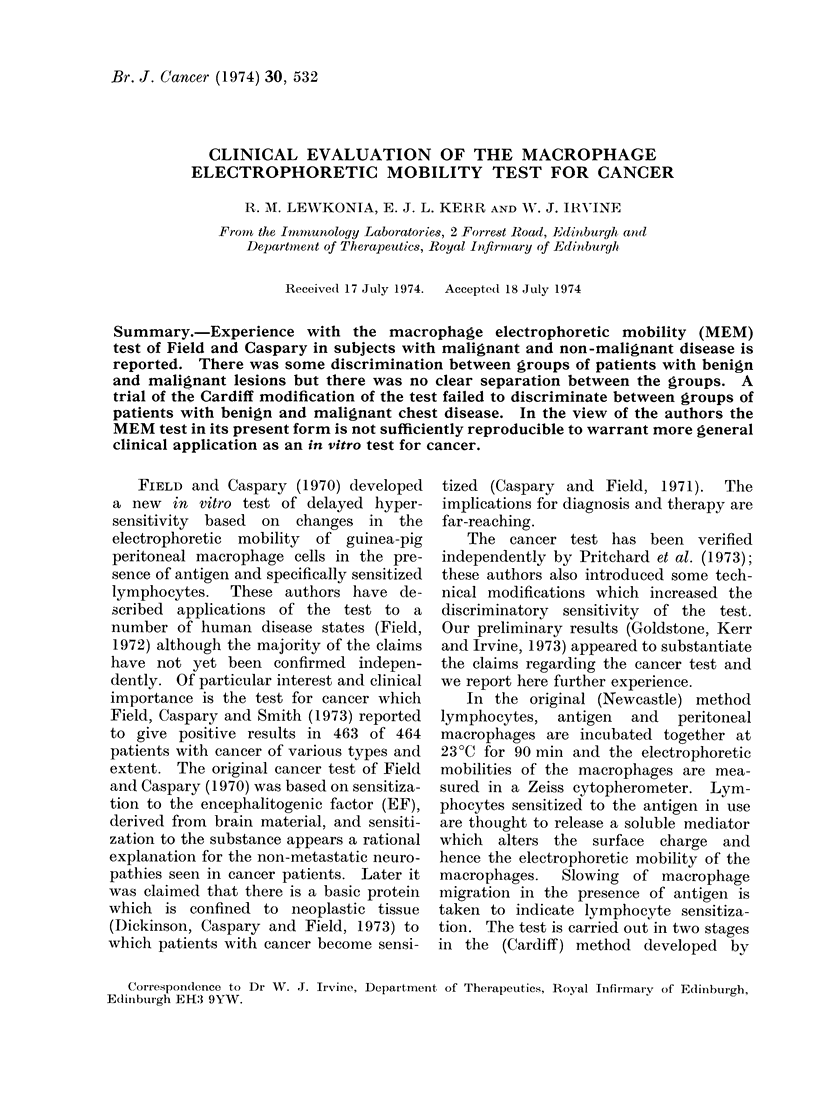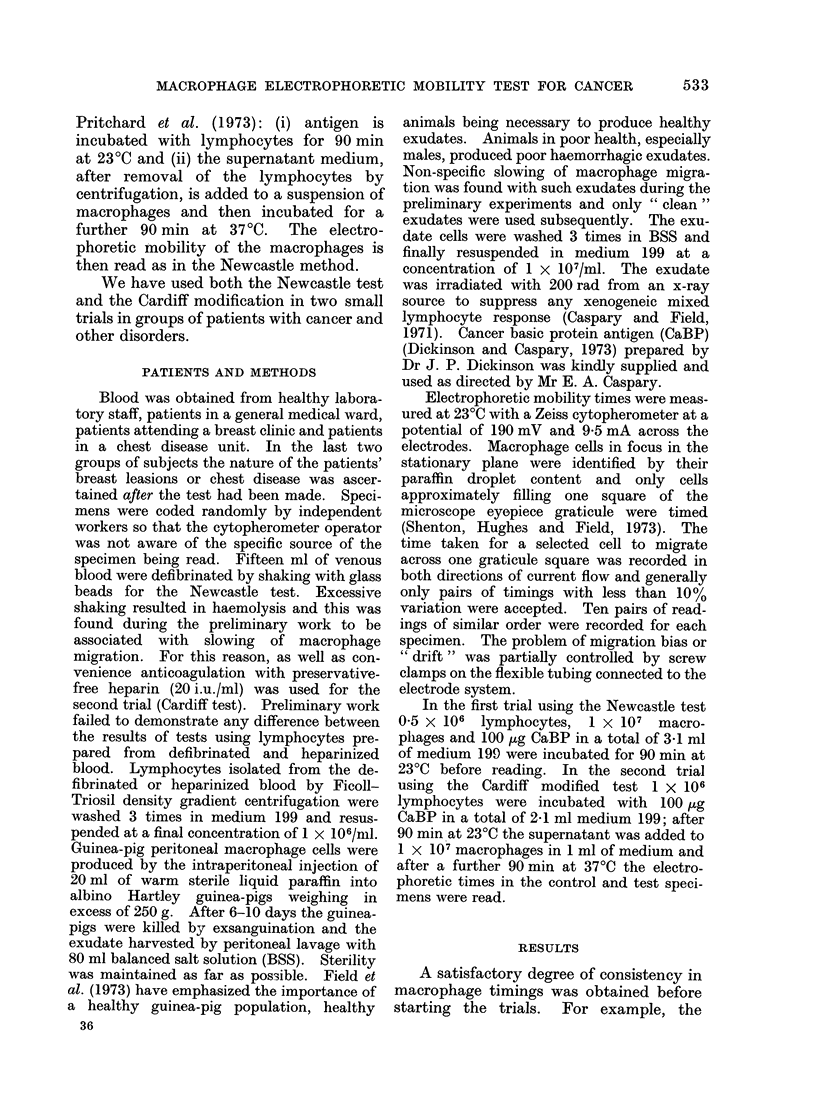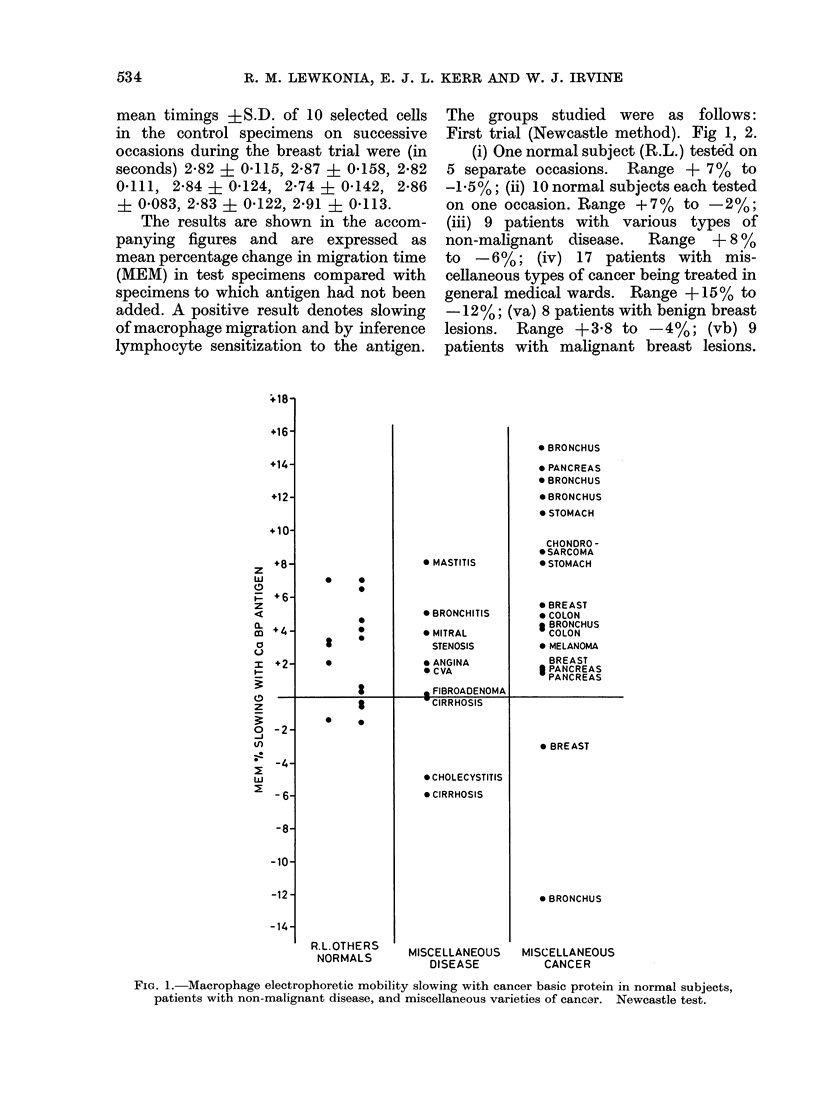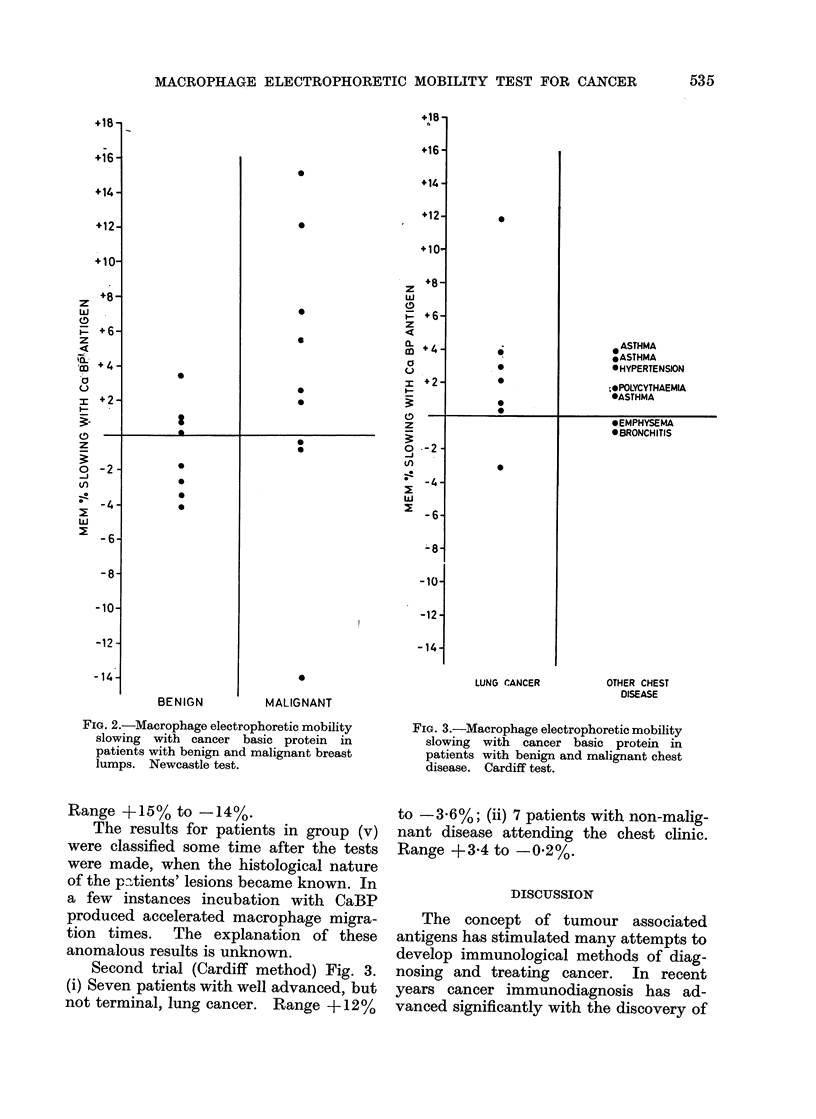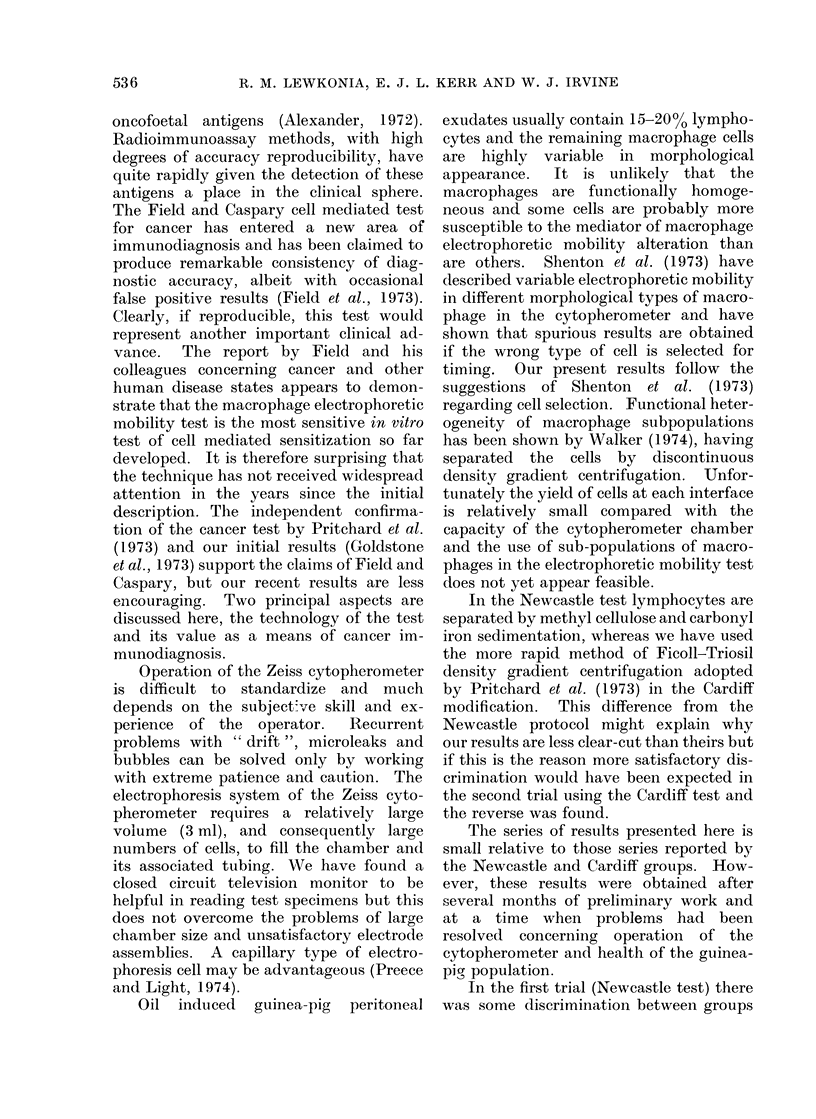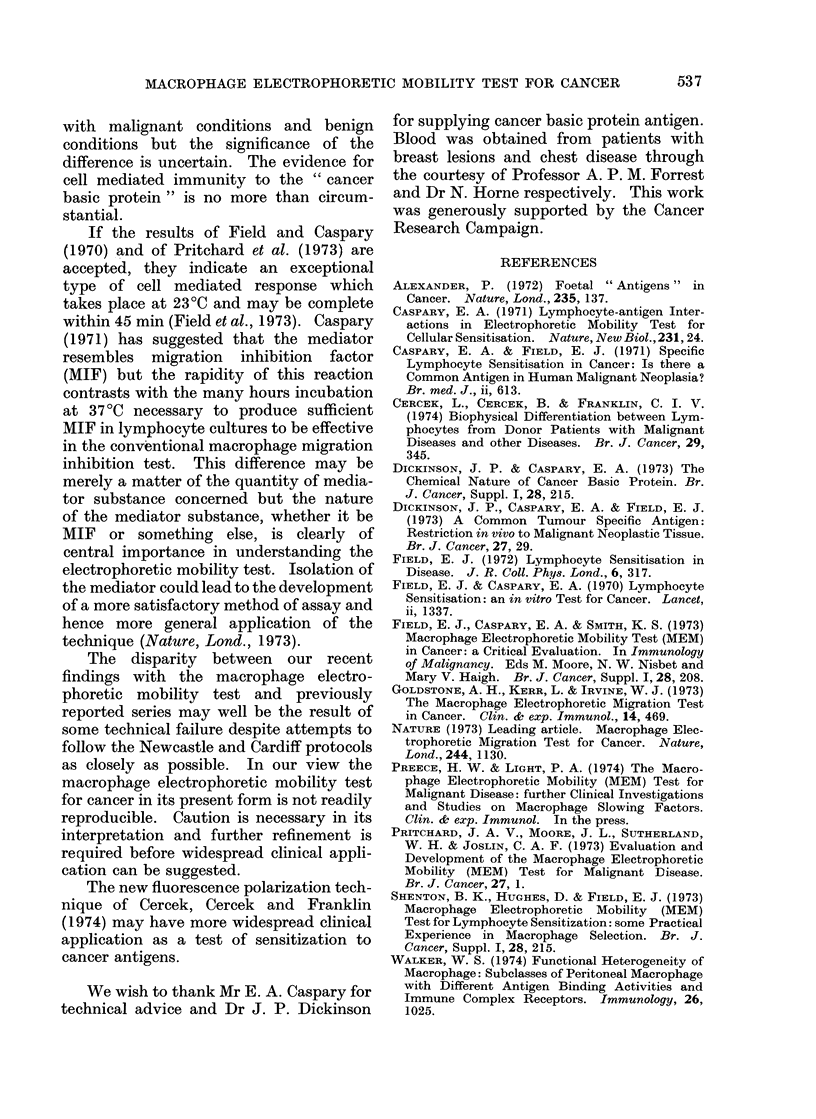# Clinical Evaluation of the Macrophage Electrophoretic Mobility Test for Cancer

**DOI:** 10.1038/bjc.1974.232

**Published:** 1974-12

**Authors:** R. M. Lewkonia, E. J. L. Kerr, W. J. Irvine

## Abstract

Experience with the macrophage electrophoretic mobility (MEM) test of Field and Caspary in subjects with malignant and non-malignant disease is reported. There was some discrimination between groups of patients with benign and malignant lesions but there was no clear separation between the groups. A trial of the Cardiff modification of the test failed to discriminate between groups of patients with benign and malignant chest disease. In the view of the authors the MEM test in its present form is not sufficiently reproducible to warrant more general clinical application as an *in vitro* test for cancer.


*Br. J. Cancer* (1974) **30**, 532

### CLINICAL EVALUATION OF THE MACROPHAGE ELECTROPHORETIC MOBILITY TEST FOR CANCER

R. M. LEWKONIA, E. J. L. KERR AND W. J. IRVINE

*From the Immunology Laboratories, 2 Forrest Road, Edinburgh and Department of Therapeutics, Royal Infirmary of Edinburgh*

Received 17 July 1974. Accepted 18 July 1974

**Summary.—Experience with the macrophage electrophoretic mobility (MEM) test of Field and Caspary in subjects with malignant and non-malignant disease is reported. There was some discrimination between groups of patients with benign and malignant lesions but there was no clear separation between the groups. A trial of the Cardiff modification of the test failed to discriminate between groups of patients with benign and malignant chest disease. In the view of the authors the MEM test in its present form is not sufficiently reproducible to warrant more general clinical application as an *in vitro* test for cancer.**

FIELD and Caspary (1970) developed a new *in vitro* test of delayed hypersensitivity based on changes in the electrophoretic mobility of guinea-pig peritoneal macrophage cells in the presence of antigen and specifically sensitized lymphocytes. These authors have described applications of the test to a number of human disease states (Field, 1972) although the majority of the claims have not yet been confirmed independently. Of particular interest and clinical importance is the test for cancer which Field, Caspary and Smith (1973) reported to give positive results in 463 of 464 patients with cancer of various types and extent. The original cancer test of Field and Caspary (1970) was based on sensitization to the encephalitogenic factor (EF), derived from brain material, and sensitization to the substance appears a rational explanation for the non-metastatic neuropathies seen in cancer patients. Later it was claimed that there is a basic protein which is confined to neoplastic tissue (Dickinson, Caspary and Field, 1973) to which patients with cancer become sensitized (Caspary and Field, 1971). The implications for diagnosis and therapy are far-reaching.

The cancer test has been verified independently by Pritchard *et al.* (1973); these authors also introduced some technical modifications which increased the discriminatory sensitivity of the test. Our preliminary results (Goldstone, Kerr and Irvine, 1973) appeared to substantiate the claims regarding the cancer test and we report here further experience.

In the original (Newcastle) method lymphocytes, antigen and peritoneal macrophages are incubated together at 23°C for 90 min and the electrophoretic mobilities of the macrophages are measured in a Zeiss cytopherometer. Lymphocytes sensitized to the antigen in use are thought to release a soluble mediator which alters the surface charge and hence the electrophoretic mobility of the macrophages. Slowing of macrophage migration in the presence of antigen is taken to indicate lymphocyte sensitization. The test is carried out in two stages in the (Cardiff) method developed by

Correspondence to Dr W. J. Irvine, Department of Therapeutics, Royal Infirmary of Edinburgh, Edinburgh EH3 9YW.

Pritchard *et al.* (1973): (i) antigen is incubated with lymphocytes for 90 min at 23 °C and (ii) the supernatant medium, after removal of the lymphocytes by centrifugation, is added to a suspension of macrophages and then incubated for a further 90 min at 37 °C. The electrophoretic mobility of the macrophages is then read as in the Newcastle method.

We have used both the Newcastle test and the Cardiff modification in two small trials in groups of patients with cancer and other disorders.

### PATIENTS AND METHODS

Blood was obtained from healthy laboratory staff, patients in a general medical ward, patients attending a breast clinic and patients in a chest disease unit. In the last two groups of subjects the nature of the patients' breast leasions or chest disease was ascertained *after* the test had been made. Specimens were coded randomly by independent workers so that the cytopherometer operator was not aware of the specific source of the specimen being read. Fifteen ml of venous blood were defibrinated by shaking with glass beads for the Newcastle test. Excessive shaking resulted in haemolysis and this was found during the preliminary work to be associated with slowing of macrophage migration. For this reason, as well as convenience anticoagulation with preservative-free heparin (20 i.u./ml) was used for the second trial (Cardiff test). Preliminary work failed to demonstrate any difference between the results of tests using lymphocytes prepared from defibrinated and heparinized blood. Lymphocytes isolated from the defibrinated or heparinized blood by Ficoll–Triosil density gradient centrifugation were washed 3 times in medium 199 and resuspended at a final concentration of $1 \times 10^6$/ml. Guinea-pig peritoneal macrophage cells were produced by the intraperitoneal injection of 20 ml of warm sterile liquid paraffin into albino Hartley guinea-pigs weighing in excess of 250 g. After 6–10 days the guinea-pigs were killed by exsanguination and the exudate harvested by peritoneal lavage with 80 ml balanced salt solution (BSS). Sterility was maintained as far as possible. Field *et al.* (1973) have emphasized the importance of a healthy guinea-pig population, healthy animals being necessary to produce healthy exudates. Animals in poor health, especially males, produced poor haemorrhagic exudates. Non-specific slowing of macrophage migration was found with such exudates during the preliminary experiments and only " clean " exudates were used subsequently. The exudate cells were washed 3 times in BSS and finally resuspended in medium 199 at a concentration of $1 \times 10^7$/ml. The exudate was irradiated with 200 rad from an x-ray source to suppress any xenogeneic mixed lymphocyte response (Caspary and Field, 1971). Cancer basic protein antigen (CaBP) (Dickinson and Caspary, 1973) prepared by Dr J. P. Dickinson was kindly supplied and used as directed by Mr E. A. Caspary.

Electrophoretic mobility times were measured at 23°C with a Zeiss cytopherometer at a potential of 190 mV and 9·5 mA across the electrodes. Macrophage cells in focus in the stationary plane were identified by their paraffin droplet content and only cells approximately filling one square of the microscope eyepiece graticule were timed (Shenton, Hughes and Field, 1973). The time taken for a selected cell to migrate across one graticule square was recorded in both directions of current flow and generally only pairs of timings with less than 10% variation were accepted. Ten pairs of readings of similar order were recorded for each specimen. The problem of migration bias or " drift " was partially controlled by screw clamps on the flexible tubing connected to the electrode system.

In the first trial using the Newcastle test $0{\cdot}5 \times 10^6$ lymphocytes, $1 \times 10^7$ macrophages and 100 $\mu$g CaBP in a total of 3·1 ml of medium 199 were incubated for 90 min at 23°C before reading. In the second trial using the Cardiff modified test $1 \times 10^6$ lymphocytes were incubated with 100 $\mu$g CaBP in a total of 2·1 ml medium 199; after 90 min at 23°C the supernatant was added to $1 \times 10^7$ macrophages in 1 ml of medium and after a further 90 min at 37°C the electrophoretic times in the control and test specimens were read.

### RESULTS

A satisfactory degree of consistency in macrophage timings was obtained before starting the trials. For example, the mean timings ±S.D. of 10 selected cells in the control specimens on successive occasions during the breast trial were (in seconds) 2·82 ± 0·115, 2·87 ± 0·158, 2·82 0·111, 2·84 ± 0·124, 2·74 ± 0·142, 2·86 ± 0·083, 2·83 ± 0·122, 2·91 ± 0·113.

The results are shown in the accompanying figures and are expressed as mean percentage change in migration time (MEM) in test specimens compared with specimens to which antigen had not been added. A positive result denotes slowing of macrophage migration and by inference lymphocyte sensitization to the antigen.

The groups studied were as follows: First trial (Newcastle method). Fig 1, 2.

(i) One normal subject (R.L.) tested on 5 separate occasions. Range + 7% to -1·5%; (ii) 10 normal subjects each tested on one occasion. Range +7% to −2%; (iii) 9 patients with various types of non-malignant disease. Range +8% to −6%; (iv) 17 patients with miscellaneous types of cancer being treated in general medical wards. Range +15% to −12%; (va) 8 patients with benign breast lesions. Range +3·8 to −4%; (vb) 9 patients with malignant breast lesions.

FIG. 1.—Macrophage electrophoretic mobility slowing with cancer basic protein in normal subjects, patients with non-malignant disease, and miscellaneous varieties of cancer. Newcastle test.

FIG. 2.—Macrophage electrophoretic mobility slowing with cancer basic protein in patients with benign and malignant breast lumps. Newcastle test.

FIG. 3.—Macrophage electrophoretic mobility slowing with cancer basic protein in patients with benign and malignant chest disease. Cardiff test.

Range +15% to −14%.

The results for patients in group (v) were classified some time after the tests were made, when the histological nature of the patients' lesions became known. In a few instances incubation with CaBP produced accelerated macrophage migration times. The explanation of these anomalous results is unknown.

Second trial (Cardiff method) Fig. 3. (i) Seven patients with well advanced, but not terminal, lung cancer. Range +12% to −3·6%; (ii) 7 patients with non-malignant disease attending the chest clinic. Range +3·4 to −0·2%.

DISCUSSION

The concept of tumour associated antigens has stimulated many attempts to develop immunological methods of diagnosing and treating cancer. In recent years cancer immunodiagnosis has advanced significantly with the discovery of oncofoetal antigens (Alexander, 1972). Radioimmunoassay methods, with high degrees of accuracy reproducibility, have quite rapidly given the detection of these antigens a place in the clinical sphere. The Field and Caspary cell mediated test for cancer has entered a new area of immunodiagnosis and has been claimed to produce remarkable consistency of diagnostic accuracy, albeit with occasional false positive results (Field *et al.*, 1973). Clearly, if reproducible, this test would represent another important clinical advance. The report by Field and his colleagues concerning cancer and other human disease states appears to demonstrate that the macrophage electrophoretic mobility test is the most sensitive *in vitro* test of cell mediated sensitization so far developed. It is therefore surprising that the technique has not received widespread attention in the years since the initial description. The independent confirmation of the cancer test by Pritchard *et al.* (1973) and our initial results (Goldstone *et al.*, 1973) support the claims of Field and Caspary, but our recent results are less encouraging. Two principal aspects are discussed here, the technology of the test and its value as a means of cancer immunodiagnosis.

Operation of the Zeiss cytopherometer is difficult to standardize and much depends on the subjective skill and experience of the operator. Recurrent problems with " drift ", microleaks and bubbles can be solved only by working with extreme patience and caution. The electrophoresis system of the Zeiss cytopherometer requires a relatively large volume (3 ml), and consequently large numbers of cells, to fill the chamber and its associated tubing. We have found a closed circuit television monitor to be helpful in reading test specimens but this does not overcome the problems of large chamber size and unsatisfactory electrode assemblies. A capillary type of electrophoresis cell may be advantageous (Preece and Light, 1974).

Oil induced guinea-pig peritoneal exudates usually contain 15–20% lymphocytes and the remaining macrophage cells are highly variable in morphological appearance. It is unlikely that the macrophages are functionally homogeneous and some cells are probably more susceptible to the mediator of macrophage electrophoretic mobility alteration than are others. Shenton *et al.* (1973) have described variable electrophoretic mobility in different morphological types of macrophage in the cytopherometer and have shown that spurious results are obtained if the wrong type of cell is selected for timing. Our present results follow the suggestions of Shenton *et al.* (1973) regarding cell selection. Functional heterogeneity of macrophage subpopulations has been shown by Walker (1974), having separated the cells by discontinuous density gradient centrifugation. Unfortunately the yield of cells at each interface is relatively small compared with the capacity of the cytopherometer chamber and the use of sub-populations of macrophages in the electrophoretic mobility test does not yet appear feasible.

In the Newcastle test lymphocytes are separated by methyl cellulose and carbonyl iron sedimentation, whereas we have used the more rapid method of Ficoll–Triosil density gradient centrifugation adopted by Pritchard *et al.* (1973) in the Cardiff modification. This difference from the Newcastle protocol might explain why our results are less clear-cut than theirs but if this is the reason more satisfactory discrimination would have been expected in the second trial using the Cardiff test and the reverse was found.

The series of results presented here is small relative to those series reported by the Newcastle and Cardiff groups. However, these results were obtained after several months of preliminary work and at a time when problems had been resolved concerning operation of the cytopherometer and health of the guinea-pig population.

In the first trial (Newcastle test) there was some discrimination between groups with malignant conditions and benign conditions but the significance of the difference is uncertain. The evidence for cell mediated immunity to the " cancer basic protein " is no more than circumstantial.

If the results of Field and Caspary (1970) and of Pritchard *et al.* (1973) are accepted, they indicate an exceptional type of cell mediated response which takes place at 23°C and may be complete within 45 min (Field *et al.*, 1973). Caspary (1971) has suggested that the mediator resembles migration inhibition factor (MIF) but the rapidity of this reaction contrasts with the many hours incubation at 37°C necessary to produce sufficient MIF in lymphocyte cultures to be effective in the conventional macrophage migration inhibition test. This difference may be merely a matter of the quantity of mediator substance concerned but the nature of the mediator substance, whether it be MIF or something else, is clearly of central importance in understanding the electrophoretic mobility test. Isolation of the mediator could lead to the development of a more satisfactory method of assay and hence more general application of the technique (*Nature, Lond.*, 1973).

The disparity between our recent findings with the macrophage electrophoretic mobility test and previously reported series may well be the result of some technical failure despite attempts to follow the Newcastle and Cardiff protocols as closely as possible. In our view the macrophage electrophoretic mobility test for cancer in its present form is not readily reproducible. Caution is necessary in its interpretation and further refinement is required before widespread clinical application can be suggested.

The new fluorescence polarization technique of Cercek, Cercek and Franklin (1974) may have more widespread clinical application as a test of sensitization to cancer antigens.

We wish to thank Mr E. A. Caspary for technical advice and Dr J. P. Dickinson for supplying cancer basic protein antigen. Blood was obtained from patients with breast lesions and chest disease through the courtesy of Professor A. P. M. Forrest and Dr N. Horne respectively. This work was generously supported by the Cancer Research Campaign.

### REFERENCES

ALEXANDER, P. (1972) Foetal " Antigens " in Cancer. *Nature, Lond.*, **235**, 137.

CASPARY, E. A. (1971) Lymphocyte-antigen Interactions in Electrophoretic Mobility Test for Cellular Sensitisation. *Nature, New Biol.*, **231**, 24.

CASPARY, E. A. & FIELD, E. J. (1971) Specific Lymphocyte Sensitisation in Cancer: Is there a Common Antigen in Human Malignant Neoplasia? *Br. med. J.*, ii, 613.

CERCEK, L., CERCEK, B. & FRANKLIN, C. I. V. (1974) Biophysical Differentiation between Lymphocytes from Donor Patients with Malignant Diseases and other Diseases. *Br. J. Cancer*, **29**, 345.

DICKINSON, J. P. & CASPARY, E. A. (1973) The Chemical Nature of Cancer Basic Protein. *Br. J. Cancer*, Suppl. I, **28**, 215.

DICKINSON, J. P., CASPARY, E. A. & FIELD, E. J. (1973) A Common Tumour Specific Antigen: Restriction *in vivo* to Malignant Neoplastic Tissue. *Br. J. Cancer*, **27**, 29.

FIELD, E. J. (1972) Lymphocyte Sensitisation in Disease. *J. R. Coll. Phys. Lond.*, **6**, 317.

FIELD, E. J. & CASPARY, E. A. (1970) Lymphocyte Sensitisation: an *in vitro* Test for Cancer. *Lancet*, ii, 1337.

FIELD, E. J., CASPARY, E. A. & SMITH, K. S. (1973) Macrophage Electrophoretic Mobility Test (MEM) in Cancer: a Critical Evaluation. In *Immunology of Malignancy*. Eds M. Moore, N. W. Nisbet and Mary V. Haigh. *Br. J. Cancer*, Suppl. I, **28**, 208.

GOLDSTONE, A. H., KERR, L. & IRVINE, W. J. (1973) The Macrophage Electrophoretic Migration Test in Cancer. *Clin. & exp. Immunol.*, **14**, 469.

NATURE (1973) Leading article. Macrophage Electrophoretic Migration Test for Cancer. *Nature, Lond.*, **244**, 1130.

PREECE, H. W. & LIGHT, P. A. (1974) The Macrophage Electrophoretic Mobility (MEM) Test for Malignant Disease: further Clinical Investigations and Studies on Macrophage Slowing Factors. *Clin. & exp. Immunol.* In the press.

PRITCHARD, J. A. V., MOORE, J. L., SUTHERLAND, W. H. & JOSLIN, C. A. F. (1973) Evaluation and Development of the Macrophage Electrophoretic Mobility (MEM) Test for Malignant Disease. *Br. J. Cancer*, **27**, 1.

SHENTON, B. K., HUGHES, D. & FIELD, E. J. (1973) Macrophage Electrophoretic Mobility (MEM) Test for Lymphocyte Sensitization: some Practical Experience in Macrophage Selection. *Br. J. Cancer*, Suppl. I, **28**, 215.

WALKER, W. S. (1974) Functional Heterogeneity of Macrophage: Subclasses of Peritoneal Macrophage with Different Antigen Binding Activities and Immune Complex Receptors. *Immunology*, **26**, 1025.